# Identifying Substructures That Facilitate Compounds to Penetrate the Blood–Brain Barrier via Passive Transport Using Machine Learning Explainer Models

**DOI:** 10.1021/acschemneuro.3c00840

**Published:** 2024-05-09

**Authors:** Lucca Caiaffa Santos Rosa, Caio Oliveira Argolo, Cayque Monteiro Castro Nascimento, Andre Silva Pimentel

**Affiliations:** Departamento de Química, Pontifícia Universidade Católica do Rio de Janeiro, Rio de Janeiro, RJ 22453-900, Brazil

**Keywords:** explainable artificial intelligence, structural alerts, LIME, XAI, XML

## Abstract

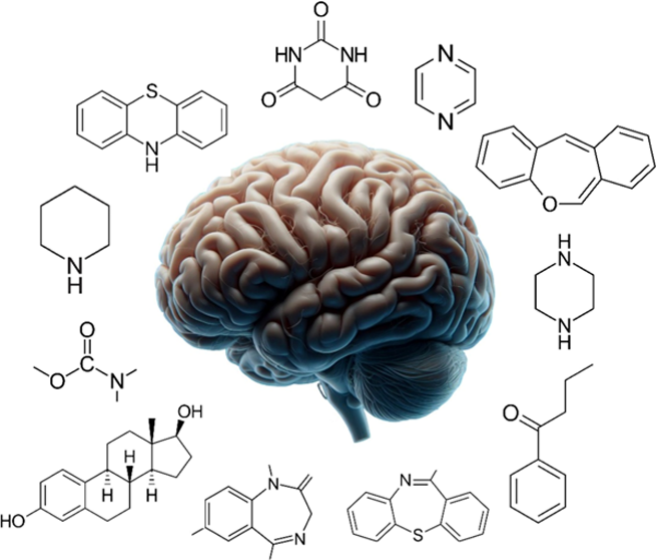

The local interpretable model-agnostic explanation (LIME) method was used to interpret two machine learning models of compounds penetrating the blood–brain barrier. The classification models, Random Forest, ExtraTrees, and Deep Residual Network, were trained and validated using the blood–brain barrier penetration dataset, which shows the penetrability of compounds in the blood–brain barrier. LIME was able to create explanations for such penetrability, highlighting the most important substructures of molecules that affect drug penetration in the barrier. The simple and intuitive outputs prove the applicability of this explainable model to interpreting the permeability of compounds across the blood–brain barrier in terms of molecular features. LIME explanations were filtered with a weight equal to or greater than 0.1 to obtain only the most relevant explanations. The results showed several structures that are important for blood–brain barrier penetration. In general, it was found that some compounds with nitrogenous substructures are more likely to permeate the blood–brain barrier. The application of these structural explanations may help the pharmaceutical industry and potential drug synthesis research groups to synthesize active molecules more rationally.

## Introduction

Currently, one of the main challenges in medicine involving central nervous system (CNS) therapy is addressing the shortage of potential drugs that can cross the blood–brain barrier (BBB).^1−7^ The BBB is a highly selective structure that protects the CNS from neurotoxic substances, bacteria, parasites, and other solutes. It is related to diseases such as meningitis,^8^ multiple sclerosis,^9^ and Alzheimer’s.^10^ In addition to the requirement of good activity, adequate metabolic properties, and low toxicity, the predictability of the blood–brain barrier permeability of an active compound is an important aspect of drug development using *in silico* techniques.^11,12^ An example of this is the search for candidates that have similar molecular and pharmacokinetic properties to lomustine, an effective anticancer agent in the treatment of brain tumors in children^13^ and other studies.^14−22^

The development of large datasets in drug discovery has further enhanced computational techniques and increased their use in assisting in high-throughput screening of molecular filters, which are fundamental in the search for new candidates. These filters help reduce in vivo and in vitro assays by focusing on using better-suited molecules for laboratory testing, saving time and money.^23−26^ Recently, machine learning (ML) methods have generated quantitative relationships between drug penetration and drug properties such as molecular weight, polar surface area, and partition coefficients.^27^ Furthermore, a classification model was developed to improve the predictability of BBB penetration in which the most common penetrating compounds and their properties were analyzed using descriptors.^28^ According to these authors, there are privileged species with substructures that are easier to penetrate through the BBB.^28^ Additionally, other studies indicate that the acidity and basicity of the compounds must be taken into account for BBB penetration, as well as aromaticity and the number of hydrogen bonding donors and acceptors.^29−31^

ML models are mostly considered “black boxes” because we do not understand which factors led to a particular prediction.^32−36^ The interpretability of these ML models is a key factor in increasing their reliability.^32,35−39^ Explainable artificial intelligence (XAI) is a method through which human beings can interpret the predictions made by an ML model, contrasting with the “black box” concept. Fortunately, explanation methods can be used so that the model prediction can be contrasted with human knowledge. Local interpretable model-agnostic explanations (LIME) is a popular example of an explanation method used to interpret ML models. As the name implies, LIME performs a single prediction on a local behavior (mostly linear) from a complex classifier with a highly nonlinear decision boundary.^32,34,40^

The contribution of the LIME method lies in its ability to provide interpretable explanations for complex machine learning models. It helps to bridge the gap between the complex inner workings of these models and human understanding by offering locally faithful explanations. This means that for a given prediction, LIME can generate an explanation that is understandable and aligned with human intuition, even if the underlying model is highly complex or nonlinear. This added value is crucial in fields where model transparency and interpretability are vital, such as in toxicology, healthcare, finance, and law, where decisions impact the lives of individuals and require justification and trust. The method is effective when working with a small set of molecular descriptors and molecule representation such as SMILES.^41^ However, it tends to fail when working with thousands of descriptors or molecular graphs,^42^ and it can convey little insight into human understanding if used inappropriately.^41^ LIME has recently been used with great ability and efficiency to find and interpret substructures from datasets of toxicology.^43^

In general, descriptors based on physicochemical properties, such as polar surface area, and hydrogen bonding acceptors and donors, are used in ML models to analyze the BBB penetration of drugs.^44^ Additionally, methods with graph convolutional networks are used to improve the understanding of the penetration mechanism, which occurs mainly by passive diffusion.^45^ The BBB penetration ability of organic structures has also been investigated with good accuracy using artificial neural networks (ANN)^46^ and graph neural networks.^47^ It is also known that random forest models have better predictive capacity to describe the BBB penetration.^48^ One of the recent discoveries of XAI in minimizing the black box effects is that these methods have the applicability to interpret the binding of chemical structures with two protein targets of pharmacological relevance using a locally interpretable model.^49^ In addition, XAI methods based on counterfactuals have already been applied to interpret the BBB penetration.^41^

XAI is the border of knowledge in the analysis of the structure–activity relationship of molecules, and interpretable models are being increasingly developed.^50−52^ In this work, the most common substructures present in the compounds that penetrate the BBB were identified using LIME. It is important to mention that this study does not underestimate the complexity of the brain, especially in terms of its pharmacokinetic properties. Then, it is worth mentioning that the most common substructures found here should not be considered as rules.^33^ However, the substructures that render compounds to penetrate the BBB should have an easier BBB penetration, making a faster CNS action and being an important factor for the discovery of new candidates for the pharmaceutical industry.^53−55^ Considering that there are few works in this area focusing on the structure of chemical compounds, it is intended to seek and verify the consistency of the models by contrasting the LIME predictions, check if they are suitable techniques to generate the substructures important to the BBB penetration, and compare with results found in the literature.

The research question addressed in the LIME method is how to provide interpretable explanations for complex machine learning models, especially in situations in which the models themselves are not inherently interpretable. LIME builds on established prior art in the field of interpretable machine learning and aims to answer this question by proposing a framework that generates explanations that are both locally faithful to the predictions of the model and understandable to humans. By doing so, LIME contributes to making machine learning models more transparent, trustworthy, and accountable in various domains. The purpose of this study is to identify substructures that facilitate the penetration of compounds through the BBB using ML models such as the deep residual network (DRN), random forest (RF), and extra trees (ET) classifier models. The quality of these models is assessed using metrics such as precision, recall, F1, and accuracy scores. The ML models are interpreted using the explanation LIME, which identifies the most significant substructures that explain BBB penetration. The interpretation of results from the LIME method can indeed be challenging, particularly in cases such as blood–brain barrier penetration and structural alert interpretation for toxicology, where domain-specific knowledge and heuristics are crucial. The reliance of LIME on local model approximations and perturbations to explain complex model predictions may lead to ad hoc or even spurious interpretations, if not carefully validated. Statistically, it is challenging to determine the causal relationship between identified substructures and blood–brain barrier penetration solely on the basis of LIME results. LIME provides a local explanation of model behavior for a specific instance but does not directly imply causality. To assess the validity of the LIME results, it is essential to complement them with domain knowledge and experimental validation. Statistical tests, such as hypothesis testing or correlation analysis, could be used to evaluate the significance of substructures identified by LIME, but these tests alone may not establish causality without additional evidence and validation. The novelty of our work lies in providing an explained comprehensive list of substructures important for BBB penetration that surpasses the existing knowledge obtained from the explainable methods so far.

## Methodology

The Python packages DeepChem (version 2.7.1.),^56^ RDKit (version rdkit-2023.9.1),^56,57^ and LIME (version lime-0.2.0.1)^34,40,58^ were installed in a Google Colaboratory platform. Other Python auxiliary packages such as mols2grid (version mols2grid-2.0.0),^59^ Matplotlib (version 3.7.1),^60^ Scikit-learn (version 1.2.2),^61^ pandas (version 1.5.3),^62^ Numpy (version 1.23.5),^63^ and IPython^64^ were also installed.

The BBBP dataset^48^ was uploaded, which makes part of the benchmark MoleculeNet dataset^65^ built for training and validating molecular machine learning models of molecular properties. This BBBP dataset incorporates binary labels (0 for nonpenetrating and 1 for penetrating molecules) on permeability properties for more than 2000 drugs, hormones, and neurotransmitters. We used DeepChem^56^ to implement various known data splitters, featurizers, transformers, machine learning classifier models, and infrastructure needed to create a classification model of compounds with blood–brain barrier penetration. The BBBP dataset^48^ was featurized using 1024 extended-connectivity fingerprints (ECFPs),^66^ which are a class of topological fingerprints for molecular characterization. Then, this featurized dataset was randomly split (80/20 split) to train and validate the models using the K-fold cross-validation method (*K* = 5) for the DRN, RF, and ET classifier models. It is important to mention that the model is randomly generated for each run. Therefore, the results may vary from each other. In this way, because it was decided not to save the model, it was possible to generate different substructures to evaluate the results from each run and to check the consistency of the results.

First, we used the DRN model (known as the MultiTaskClassifier model from the DeepChem library).^56^ The MultiTaskClassifier model is a fully connected deep residual network for multitask classification composed of preactivation residual blocks. Hyperparameterization for this first model was performed to optimize the best parameters, including layer size [256, 256], [256, 256, 256], [256, 256, 256, 256], dropout [0.0, 0.1], learning rate [0.005, 0.0035, 0.0025, 0.0010], number of epochs [5, 10, 20, 30, 40, 50], momentum [0.0, 0.9], decay [0.0, 0.1], and batch size [32, 64, 128]. This was done using the GridHyperparamOpt optimizer,^67^ the hyperparameter search method,^61^ and the ROC-AUC metric.^68^ Later, we also used the RF and ET classifier models from the Scikit-learn library.^61^ The hyperparameterization for these latter models was done by optimization of the number of estimators (50 and 500) and maximum depth (1 and 20) of the trees using the randomized search CV method^61^ and the ROC-AUC metric.^68^ Then, the best models were used in the next steps with the training and validation datasets using the area under the ROC curve (ROC-AUC metric). The results were analyzed with the confusion matrix, accuracy, precision, F1, and recall scores.

After creating the model using the training dataset and analyzing it with the validation dataset using the aforementioned statistical analysis described below, the LIME package^34,40,58^ was used to explain the model. Once the model is generated, LIME uses the LimeTabularExplainer function in classification mode to explain the training data. The function takes in categorical names and feature names, which are represented as dictionaries and lists of indexes, respectively. This creates an explainer object for LIME, which uses 1024 circular fingerprints to represent the training dataset and feature names. The model predicted and explained the validation dataset using 100 features. The explainer object then returns a dictionary mapping the fingerprint index to the list of SMILES strings that activated that fingerprint. Since our features do not have natural names, they are simply numbered in the feature_names object. The class names in the BBBP dataset represent brain–blood barrier penetration assays and are binary labels indicating penetration or nonpenetration. ″0″ represents “no penetration” and ″1″ represents “penetration.”

The evaluation is performed for each molecule to explain why the model predicts whether a molecule will penetrate the brain–blood barrier or not. The model takes a 2D numpy array (samples, features) as input and returns predictions for each sample. The output is stored in a model function object. By applying a threshold of 0.8, we obtain a list of penetrating molecules in the validation dataset that are correctly predicted to penetrate. The task is then to loop through the list of molecules in the validation dataset, evaluate the model, and store the results in an object. This list, along with the object, is input into the explain instance class to determine why a molecule was predicted to penetrate. The explainer identifies the most sensitive features to the prediction by analyzing the elements in the fingerprint that correspond to one or more fragments. The output is stored in the explainer object, which contains information about which fragments contributed to the prediction.

The LIME explanation module provides visualization and mapping functions for the explainers. The as_map() class generates a map of explanations with labels, represented as a list of tuples containing the feature identification number and its weight. This class is used to obtain the information in a more suitable format for processing. The keys in this map are the labels, and the value for each key is a list of tuples containing the fingerprint index and its respective weight. This is then converted into a dictionary, mapping indices to weights. In the my_fragments object, the fragments are present for each molecule of interest, while the fragment_weights object (fragment_weight = dict(exp.as_map()[1])) contains information about which fragments contributed to the prediction.

The weights assigned by LIME represent the contribution of a fragment in a molecule to the prediction, ranging from 0 to −1 or +1. However, the sum of these weights for each molecule does not necessarily equal 1. These weights still indicate the contribution of a fragment to the prediction. Each molecule has multiple fragments, and each fragment can contribute positively or negatively to the prediction of penetration. It is possible for a molecule to have some fragments with positive contributions and others with no contribution. Therefore, the model classifies a molecule as “penetrating” or “nonpenetrating” by separately summing all of the positive and negative contributions. If the sum of the positive weights is greater than the sum of the negative weights, the molecule is classified as “penetrating.” Conversely, if the sum of the negative weights is greater than the sum of the positive weights, the molecule is classified as “nonpenetrating.”

At the end, the classification data of each active molecule for BBB penetration is exported. The number of occurrences with which a given substructure is obtained in the prediction of BBB penetration of molecules and their respective total weight of contribution. For each of these molecules, dictionaries are obtained with the counting frequency of each substructure and the total sum of associated weights for each substructure. Then, these dictionaries are converted in such a way that the desired data frame is obtained. Finally, RDKit resources are used to highlight the substructures, and their visualization in the associated compounds is rendered by mols2grid^59^ and Pandas libraries.^62^

LIME is versatile and applicable to any ML model. It is characterized as independent, easy to comprehend, and didactic.^34,40,58,69^ LIME aims to elucidate a representative set of model predictions and ensure local reliability by replicating model behavior with fidelity on a smaller scale. The key feature of LIME is its ability to construct a linear model in the proximity of a test instance. This involves initially generating artificial samples by permuting such instances with weights determined by a kernel value. Consequently, the model is trained to make predictions based on the importance coefficients of the interpreted characteristics of the model. When the LIME explainer module is utilized, it is noteworthy that testing various kernel width values did not yield unstable results. Moreover, the substructures produced in the explanation remained largely consistent across different kernel values.^43^ Consequently, the default value recommended by the LIME developers was adopted. To enhance the interpretability of results, efforts were made to eliminate unimportant substructures for BBB penetration. For conciseness, certain substructures with small or negative weights were excluded by filtering all fragments with a negative weight. In essence, what dictates the behavior in a drug is the substructure that facilitates BBB penetration, justifying the use of this cutoff.

## Results

### Data Curation

The SMILES representations in the benchmark dataset have been rectified manually, addressing an issue where 11 SMILES entries in the BBB dataset depicted uncharged tetravalent nitrogen atoms. It is essential to note that tetravalent nitrogen atoms should invariably bear a charge. The presence of these errors rendered the chemical structures unreadable by RDKit. Despite the widespread use of this dataset in numerous studies, there is a noticeable absence of discussions regarding the handling of these invalid SMILES representations. To ensure the integrity of chemical structures, the SMILES representations were meticulously corrected and the requisite positive charges were introduced where necessary in the benchmark datasets.

Depending on the structure, the carboxylic acid substructure in some molecules is represented in one of three different forms: the protonated acid, the anionic carboxylate, and the anionic salt form. For the sake of simplicity, the form used in this study was neutral, as it should be for most structures in physiological pH.

In the BBB dataset, 81 structures were identified as duplicates and 7 as triplicates, which is undesirable for a benchmark. To address this issue, the authors systematically removed these duplicate/triplicate compounds to prevent any potential issues. Remarkably, the BBB dataset includes 14 compounds (13 pairs and 1 trio), where the identical molecules are inconsistently labeled as, for example, BBB penetrant and BBB nonpenetrant at the same time. That is why these inconsistencies were removed. It is important to mention that some of these inconsistencies were found because the molecules are treated as P-gp transported on one label and as passively transported on the other label. Other inconsistencies may come from disagreements of results by different laboratories.

### Hyperparameter Optimization

Initially, the architecture of the DRN classifier method was explored with layer sizes of [64], [128], [256], [512], and [1024]. The best result was achieved with a layer size of [256]. Then, the architecture was optimized with layer sizes of [[256, 256], [256, 256, 256], and [256, 256, 256, 256]]. Additionally, the other parameters mentioned in the Methodology Section were optimized using the procedure described in that section. The hyperparameter optimization of the DRN, RF, and ET classifier models was investigated to find the best parameters. The optimized hyperparameters are presented in Tables 1 and 2. The optimum parameters for the DRN, RF, and ET models varied with each run. Using these optimum parameters, the ROC-AUC metrics for each classification model were mostly found to be higher than 0.90 for the training and validation datasets, as shown in Table 3. Therefore, there is no indication of substantial overfitting. Although the training scores for the DRN, RF, and ET models were mostly higher than 0.98, the validation scores of the RF model are slightly higher than those of the DRN and ET models, as presented in Table 3.

**Table 1:** Optimized Parameters in the Hyperparameterization Process for the Deep Residual Network (DRN) Classifier Model in Three Different Runs (#1, #2, and #3) for the Blood–Brain Barrier Permeation of Compounds

run	layer size^a^	dropout	learning rate	epochs	momentum	decay	batches
#1	4	0.1	0.0025	50	0.9	0.1	128
#2	4	0.0	0.005	30	0.0	0.0	128
#3	4	0.1	0.001	10	0.0	0.0	128

a Layer size: 2 for [256, 256], 3 for [256, 256, 256], and 4 for [256, 256, 256, 256].

**Table 2:** Optimized Parameters in the Hyperparameterization Process for the Random Forest (RF) and Extra Trees (ET) Classifier Models in Three Different Runs (#1, #2, and #3) for the Blood–Brain Barrier Permeation of Compounds

model	run	depth	estimators
RF	#1	19	89
	#2	19	387
	#3	18	327
ET	#1	19	362
	#2	18	429
	#3	18	92

**Table 3:** Metrics (Mean ROC-AUC) of the Training and Validation Datasets for the BBB Penetration Model for Three Different Runs (#1, #2, and #3) Using the Deep Residual Network (DRN), Random Forest (RF), and Extra Trees (ET) Classifier Models for the Blood–Brain Barrier Permeation of Compounds

model	run	training	validation
DRN	#1	0.999	0.894
	#2	0.976	0.790
	#3	0.989	0.882
RF	#1	0.999	0.931
	#2	0.999	0.943
	#3	0.999	0.936
ET	#1	0.999	0.913
	#2	0.999	0.925
	#3	0.999	0.915

**Figure 1:**
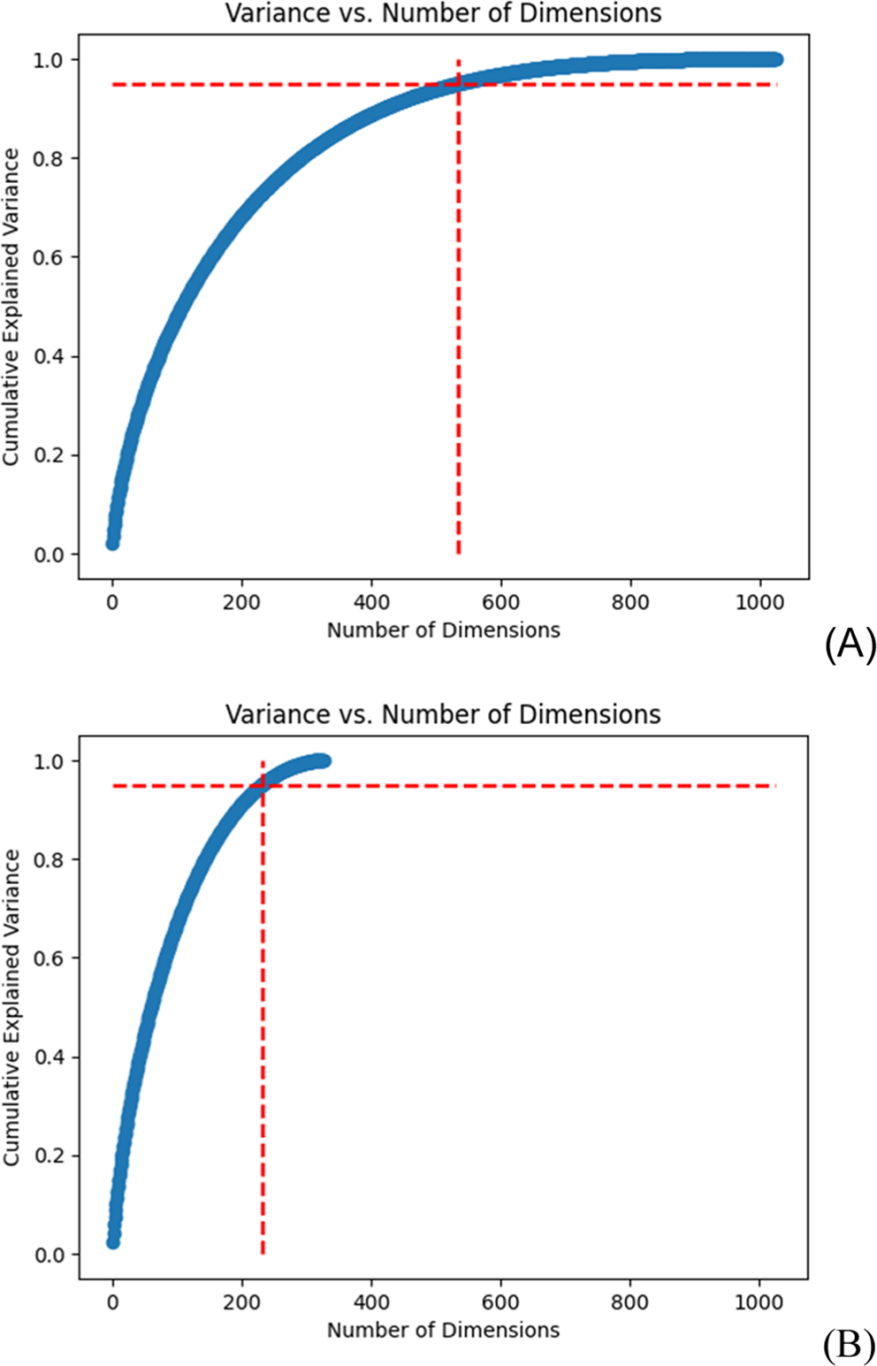
Principal component analysis of the BBB penetration dataset representing the 542 and 232 fingerprints (vertical dashed line in red) that are significant within 95% cumulative explained variance (horizontal dashed line in red) for all samples in the training and validation datasets, respectively. The number of fingerprints used in the featurization is 1024, representing the total dimensionality of the training dataset.

The parameters of the LIME explainer were thoroughly investigated. The number of features parameter, which defines the maximum number of features within a single explanation, was scrutinized. Despite the classifier model containing 1024 features, less than a dozen proved to be significant for each sample. Modifying this parameter from 20 to 500 had no discernible impact on the results or execution time. However, a principal component analysis for all samples showed that 542 and 232 fingerprints out of the total 1024 fingerprints used in the featurization were significant, representing 95% of the cumulative explained variance in the training and validation datasets, respectively, as presented in Figure 1. It was found that the results from 542 and 1024 fingerprints were similar. Initially, a conservative setting of 100 features was chosen for safety. Another parameter studied was the number of explanations with the highest probability of prediction for each substructure. This parameter ranged from zero (none) to 10. Increasing this value resulted in more refined results due to increased complexity in the explanations. However, it was observed that higher values reduced the number of substructures with weights greater than 0.1. Consequently, a decision was made to set the number of explanations to 1, maximizing the quantity of substructures. Notably, high values for the number of explanations correlated with prolonged evaluation times, exceeding 1–2 h. This underscores the importance of avoiding excessively high values to maintain reasonable processing times.

### Statistical Analysis

The precision score is the ratio TP/(TP + FP), and the recall score is the ratio TP/(TP + FN), where TP is the number of true positives, FP is the number of false positives, and FN is the number of false negatives. False Positive (FP) and True Positive (TP) instances occur when the model predicts a positive data point incorrectly and when the model accurately predicts a positive data point, respectively. On the other hand, True Negative (TN) and False Negative (FN) instances occur when the model accurately predicts a negative data point and when the model incorrectly predicts a negative data point, respectively. The F1 score is the harmonic mean of the precision and recall. It is important to mention that precision, recall, and F1 scores reach their best values at 1 and worst values at 0. While the precision score is intuitively the ability of the classifier not to label a sample that is negative as positive, the recall score is intuitively the ability of the classifier to find all of the positive samples. Accuracy is a metric used to evaluate the performance of classification models. It is the number of correct predictions as a percentage of the number of observations in the dataset. The precision, recall, F1 scores, and accuracy scores for the classifier models used in this study for the validation dataset are presented in Table 4. The detection of a substructure falsely identified as irrelevant can happen when the local explanation is not accurate in relation to the underlying behavior of the model. This behavior can occur due to various reasons, such as inadequate sampling of data or features, model complexity, or the presence of data noise. If researchers want to check the relevance and importance of the substructures identified through LIME in the context of blood–brain barrier permeation to gain more confidence, they can utilize the following validation strategies. First, we compare the identified substructures with the expertise of professionals in the field of blood–brain barrier permeation to ensure their biological plausibility and relevance. Second, experiments were conducted using existing data to validate the effects of the identified substructures on blood–brain barrier permeation. Third, statistical tests were applied to assess the significance of the identified substructures in predicting blood–brain barrier permeation. Fourth, sensitivity analysis was performed to determine if the identified substructures remain relevant under different data perturbations. Finally, we evaluate the impact of the identified substructures on model predictions by comparing the performance of the model with and without them.

**Table 4:** Precision, Recall, F1, and Accuracy Scores, and the Matthew Correlation Coefficient (MCC) for the Deep Residual Network (DRN), Random Forest (RF), and Extra Trees (ET) Classifier Models in Three Different Runs (#1, #2, and #3) for the Validation Dataset of Blood–Brain Barrier Permeation of Compounds

			scores				
models		run	precision	recall	F1	MCC	accuracy
DRN		#1	0.868	0.868	0.868	0.645	0.868
		#2	0.896	0.896	0.896	0.689	0.896
		#3	0.885	0.885	0.885	0.660	0.885
RF		#1	0.908	0.956	0.893	0.696	0.893
		#2	0.900	0.954	0.883	0.653	0.883
		#3	0.899	0.963	0.891	0.688	0.891
ET		#1	0.895	0.945	0.878	0.672	0.878
		#2	0.906	0.954	0.888	0.670	0.888
		#3	0.909	0.964	0.898	0.699	0.898

**Figure 2:**
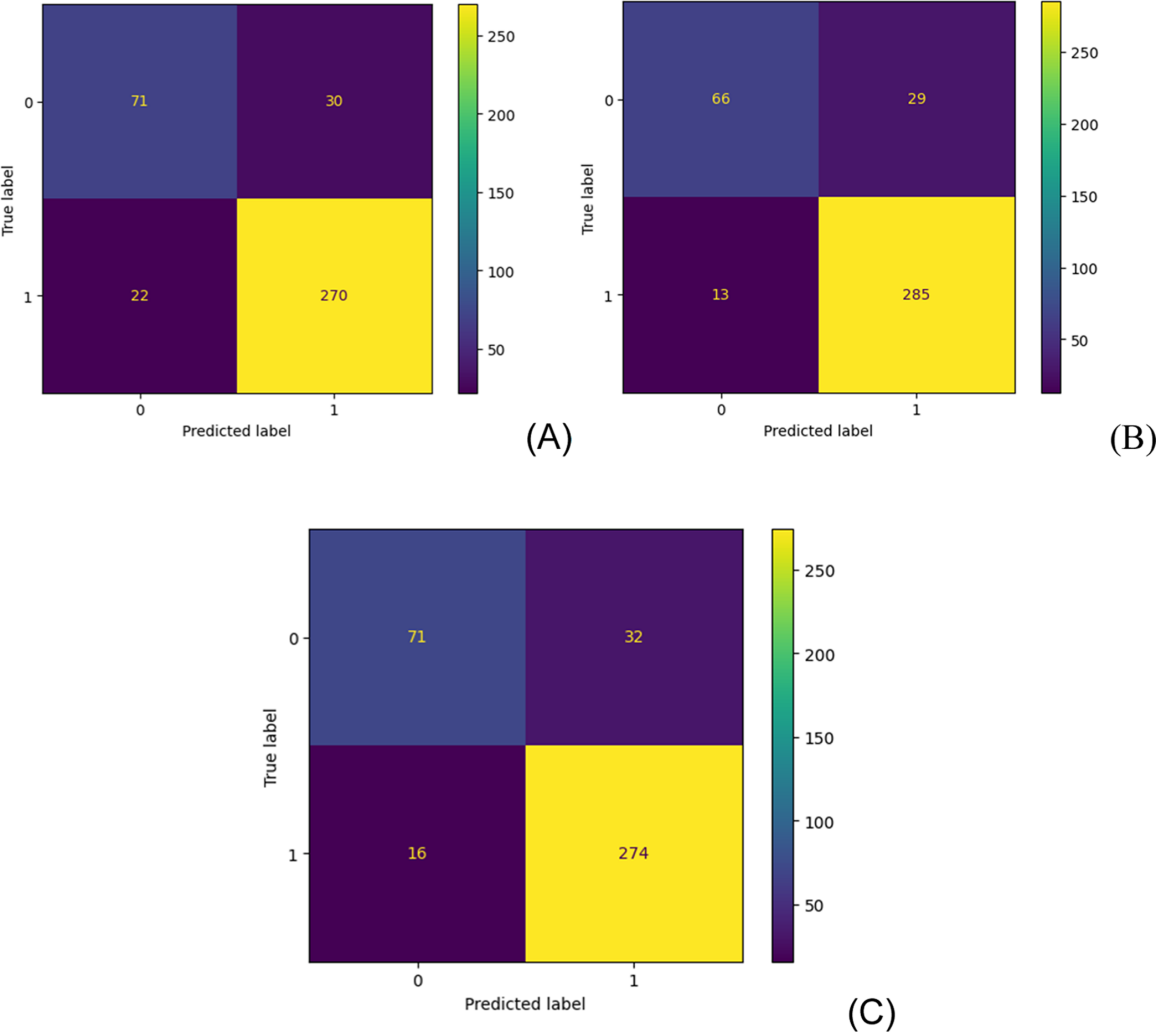
Confusion matrix for the DRN (A), RF (B), and ET (C) classification models using the validation dataset of the BBB permeation for the run #1.

As can be observed, both classifier models perform excellent classification for BBB penetrating compounds and poorer classification for BBB nonpenetrating compounds. It is also important to note that the RF and ET models are more accurate and precise than the DRN model. The confusion matrix is used to evaluate the accuracy of classification, as presented in Figure 2. By definition, a confusion matrix C is such that C*_ij_* is equal to the number of observations known to be in group I and predicted to be in group j. Thus, the count of TN is C_00_, FN is C_10_, TP is C_11_, and FP is C_01_, as presented in Figure 2 for the DRN, RF, and ET models in the validation dataset of BBBP. The TP values are large, and the TN values are small, meaning that the classification model accurately classifies the penetrating compounds in the BBB but not the nonpenetrating compounds. It is important to mention that the FP and FN values are also low, meaning that the classification model does not produce many false classifications. Of course, there are many more penetrating compounds in the BBBP validation dataset, so the classification model should only be used to classify the penetrating compounds in the BBB, and the classification for the nonpenetrating compounds is not sufficiently accurate for this purpose. It is noteworthy that a different classification model must be built using the nonpenetrating compounds if one would like to classify the nonpenetrating compounds in the BBB. In this situation, the C_00_ element in the confusion matrix would be much larger than the C_11_ element.

It is not our intention to compare whether any model or metric is better than another because of the pitfalls of comparing classification models and performance metrics solely based on statistics. The standard deviation is highlighted as a measure of variability rather than a statistical test. It quantifies the amount of variation or dispersion in a set of values. While it provides useful information about the spread of data, it is not a statistical test itself. Methods such as the Student’s *t* test and analysis of variance (ANOVA) are parametric statistical tests. These tests have assumptions about the distribution of data (e.g., normality and independence) that may not always hold true in real-world scenarios. To address the limitations of parametric tests, it is recommended to use nonparametric alternatives. The Student’s *t* test can be replaced by the Wilcoxon rank-sum test, which is a nonparametric analogue suitable for comparing two methods. For comparing more than two methods, the ANOVA is suggested but with caution about its limitations. Instead, it is advocated to use Friedman’s test, a nonparametric alternative to ANOVA, which is particularly useful when dealing with data that is not independent or normally distributed. Therefore, it is important to consider the distribution and nature of the data when comparing classification models and performance metrics. It is suggested to move away from relying solely on standard deviation and parametric tests, opting for nonparametric alternatives like the Wilcoxon rank-sum test and Friedman’s test for more robust comparisons in various scenarios. As the results of the models and metrics used in our work were quite satisfactory, it is also believed that it is not necessary to perform any statistical comparisons to determine which model or metric is better in comparison to each other.

The field of interpretability in machine learning models can progress methodologically in several key steps. First, there is a need to advance model-agnostic approaches in order to offer interpretable explanations to a more diverse group of models and datasets. Second, the integration of domain-specific knowledge into interpretability methods is essential, particularly in intricate domains such as toxicology, to enhance the relevance and accuracy of explanations. Third, developing methods to quantify uncertainty linked with interpretable explanations is crucial for providing users with a nuanced comprehension of model predictions. Fourth, enhancing the scalability and efficiency of interpretability methods is imperative for handling large datasets and complex models promptly. Finally, establishing standardized validation and benchmarking procedures is critical for evaluating the performance of interpretability methods and comparing their efficacy across various domains and tasks. By tackling these methodological challenges, the field can progress toward more dependable, interpretable, and credible machine learning models for predicting blood–brain barrier permeation.

### Model Explanation

The LIME explanations for the BBB penetration are presented below. The results were filtered with weights of >0.1 to remove less important substructures. Then, LIME selected the ninth to 10th most influential substructures for the BBB penetration. In general, LIME mostly explains the penetrability of compounds by using structures with nitrogen. Basicity is considered one of the main factors for CNS penetrability, especially for groups with nitrogen because they can be protonated at physiological pH (such as piperidine and piperazine groups). For BBB penetration, the C=N double bond of the aromatics is also considered. LIME detected the positive weight of this feature in the contribution to BBB penetration. Aromatic rings tend to facilitate penetration into the BBB because the hydrophobicity is enhanced.

**Figure 3:**
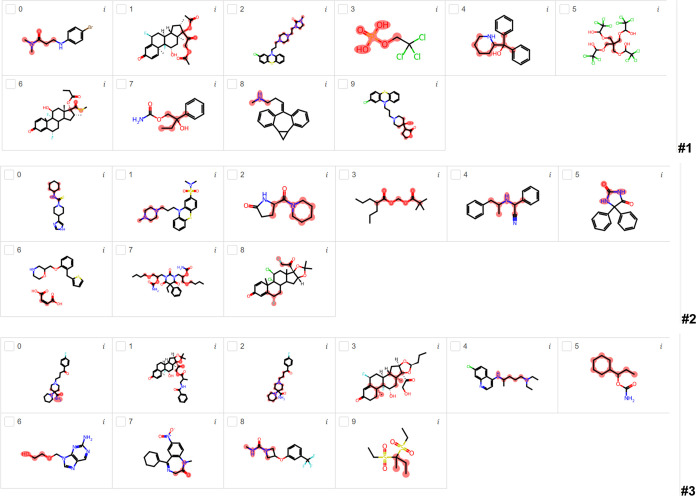
Most important substructures found by LIME for BBB penetration using the DRN classification model for runs #1, #2, and #3.

Figure 3 shows the most important substructures found by LIME for BBB penetration using the DRN classification model for runs #1, #2, and #3. From run #1, six compounds have nitrogen-containing groups (mostly cyclic and noncyclic amine groups), but only three of them are highlighted in the nitrogen group. In the other three compounds, LIME highlighted the neighborhood of the nitrogen group. However, the highlighted substructures in compounds 1 and 6 are oxygen- and sulfur-containing groups, respectively, although nonhighlighted fluorine-containing groups are found in these two molecules. Compounds 3 and 5 have nonhighlighted chlorine-containing groups, with the first one having a highlighted phosphorus-containing group. In run #2, seven compounds have nitrogen-containing groups, and five of them have highlighted nitrogen-containing groups. Although compound 6 is not highlighted in the nitrogen-containing group, compound 7 is highlighted in the nitrogen-containing group neighborhood. Compounds 3 and 8 have been highlighted as oxygen- and halogen-containing groups, respectively. In run #3, eight compounds have nitrogen-containing groups, but three of them are not highlighted in the nitrogen group. Compounds 1 and 3 have fluorine-containing groups, but the highlights are in the oxygen-containing groups. Compounds 0 and 2 are repetitions with highlights in different nitrogen-containing groups. Compounds 5 and 6 are highlighted in the hydrocarbon part of these molecules, but the nitrogen-containing groups are not highlighted in these molecules. Compound 9 is also highlighted in the hydrocarbon part of the molecule with two nonhighlighted sulfur-containing groups.

**Figure 4:**
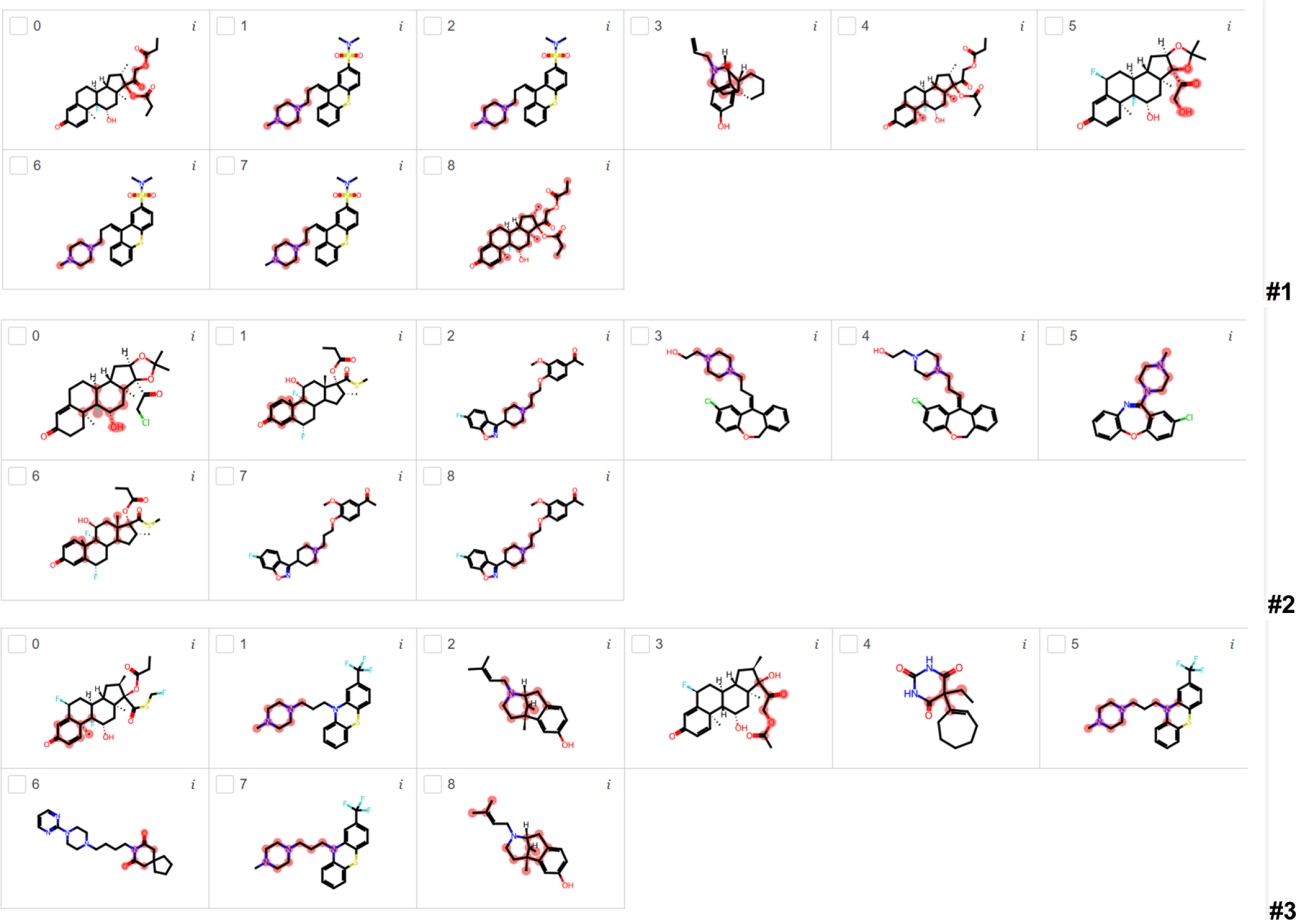
Most important substructures found by LIME for BBB penetration using the RF classification model for runs #1, #2, and #3.

Figure 4 presents the most important substructures found by LIME for BBB penetration using the RF classification model for runs #1, #2, and #3. In run #1, five compounds are highlighted with nitrogen-containing groups. Four of them (1, 2, 6, and 7) are repetitions with very similar highlighted nitrogen-containing substructures. Compounds 0, 4, and 8 are also repetitions but with different highlighted oxygen-containing groups. Compound 5 has highlighted substructures with oxygen-containing groups. In run #2, six nitrogen-containing substructures are highlighted in different compounds. Compounds 3 and 4 are repetitions with quite similar highlights, and compound 5 is a very similar compound with a highlighted nitrogen-containing group. Compounds 2, 7, and 8 are also repetitions with quite similar highlights. Compounds 1 and 6 are also repetitions with different highlights in the cyclic substructure with oxygen- and fluorine-containing groups. Compound 0 is a repetition of compound 5 in run #1 but with similar highlights (in the same region). In run #3, seven nitrogen-containing compounds are found by LIME, but two of them are highlighted in the nitrogen-containing neighborhood. The other five compounds have highlighted nitrogen-containing groups. Compounds 0 and 3 are oxygen- and fluorine-containing compounds with different highlights. Compounds 4 and 8 contain nonhighlighted nitrogen-containing substructures; however, the highlight is in the neighborhood. Compounds 5 and 7 are repetitions with very similar highlighted nitrogen-containing substructures.

**Figure 5:**
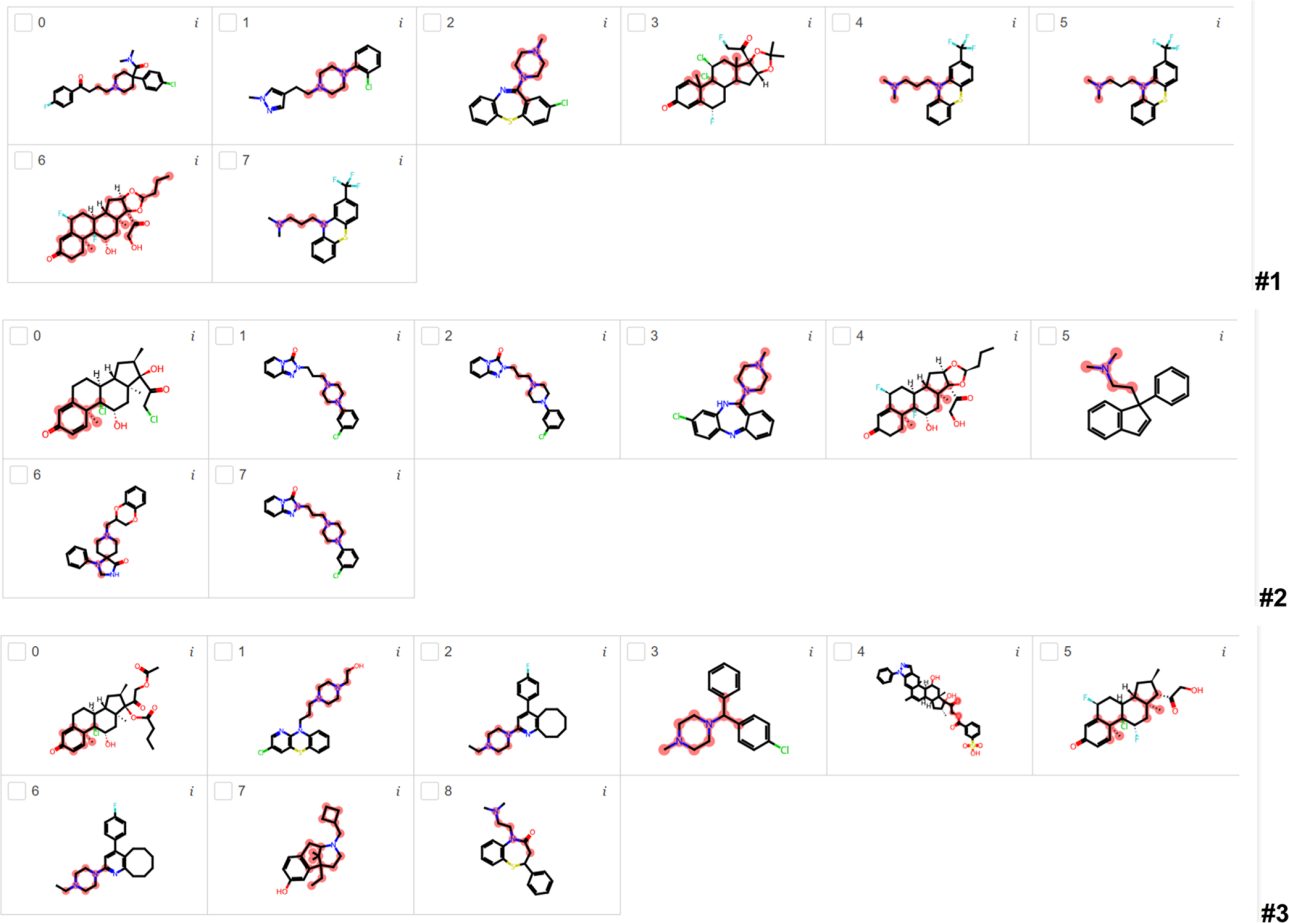
Most important substructures found by LIME for BBB penetration using the ET classification model for runs #1, #2, and #3.

Figure 5 shows the most important substructures found by LIME for BBB penetration by using the ET classification model. In run #1, six nitrogen-containing substructures are highlighted in different compounds. Compounds 4, 5, and 7 are repetitions with very similar highlighted substructures. Compounds 3 and 6 have oxygen- and halogen-containing groups with different non-nitrogen-containing highlighted substructures. In run #2, six compounds have highlighted nitrogen-containing substructures; however, compounds 1, 2, and 7 are repetitions with slightly different highlights. Compounds 0 and 4 are very different oxygen- and halogen-containing compounds with very different highlighted substructures. In run #3, seven nitrogen-containing compounds are highlighted with different substructures; however, five of them are nitrogen-containing substructures. Compounds 4 and 7 have non-nitrogen-containing highlighted substructures, but the latter one has a nitrogen-containing group in the neighborhood.

## Discussion

The permeability of chemical compounds through the BBB is a complex interplay of several factors. Understanding the significance of the functional groups is crucial. Although lipophilicity plays a pivotal role in determining the ability of BBB penetration, different functional groups interact with the BBB components, influencing the BBB permeation of a compound. However, variations in lipophilicity caused by different functional groups impact the transport of chemical compounds by influencing the solubility of the chemical compounds in the lipid-rich membranes of the barrier. Generally, hydrophobic compounds tend to have a better BBB permeation. Nevertheless, optimal ranges of lipophilicity for efficient BBB permeation depend on the specific characteristics of the functional groups of compounds. Therefore, too hydrophilic compounds may struggle for BBB penetration, while excessively lipophilic compounds may face challenges related to clearance and transport.

Indeed, the permeability of chemical compounds through the BBB is intricately influenced by specific nitrogen-containing functional groups. Therefore, several key functional groups have been identified for their potential to enhance BBB permeability. For example, amino groups (−NH_2_, −NRH, and −NR_1_R_2_) in a compound can participate in interactions, affecting its ability for BBB penetration. Chloroquine (compound 4 in DRN model run #3, Figure 3) is an aminoquinoline used for the prevention and therapy of malaria. It attenuates neuroinflammation. LIME found that the quinoline substructure does not cause BBB penetration; however, the two amine groups in the side chain are highlighted as potential substructures that enhance BBB penetration. LIME also found the amfetaminil drug (compound 4 in DRN model run #2, Figure 3), a stimulant drug derived from amphetamine that has an amine group. Another example is octriptyline (compound 8 in DRN model run #1, Figure 3), a tricyclic antidepressant, and thioperamide (compound 0 in DRN run #2, Figure 3), used to prevent seizures or reduce their severity. Finally, tiazesim (compound 8 in ET model run #3, Figure 5) is a heterocyclic antidepressant related to tricyclic antidepressants. In this molecule, LIME highlighted the tertiary amine group in the side chain linked to the heterocyclic tertiary amine substructure.

Carbamate group (>N–C(=O)–O−) has unique properties that may also influence BBB permeability. Their involvement in hydrogen-bonding interactions with BBB components can impact the overall transport process. Oxyfenamate is a sedative and anxiolytic drug of the carbamate class found by LIME (compound 7 in DRN model run #1, Figure 3). Procymate (compound 5 in DRN model run #3, Figure 3) is a carbamate derivative that is a sedative and anxiolytic drug. Also, difebarbamate is a tranquilizer of the barbiturate and carbamate families found by LIME (compound 7 in DRN model run #2, Figure 3), but it only highlighted the carbamate substructure, not the barbiturate substructure. On the other hand, LIME highlighted part of the barbiturate ring of heptabarbital (compound 4 in RF model run #3, Figure 4), which is a sedative and hypnotic drug of the barbiturate family.

Nitrogen-containing heterocycles (such as imidazole) are known for their potential to enhance permeability. The combination of the aromatic nature of these structures, with their ability to participate in various interactions, contributes to their favorable influence on BBB permeation. It is important to emphasize that many compounds presented in Figures 3–5 are amines, amides, and nitrogen-containing heterocycles. In the following, some noteworthy examples of nitrogen-containing heterocycles exhibiting distinctive properties that affect the permeability of chemical compounds through the BBB are shown.

Pyrrole or pyrrolidine rings feature a nitrogen atom in a five-membered ring that can engage in various interactions with BBB components, potentially influencing transport and impacting BBB permeability. Piperidine and piperazine (and pyrazine) consist of six-membered rings with one nitrogen atom and two nitrogen atoms at opposite positions in the ring, respectively. Their unique structural characteristics may contribute to their potential influence on BBB permeation. LIME found the piperidine ring in pipradrol (compound 4 in DRN model run #1, Figure 3), a psychoactive agent and a central nervous system stimulant that has proven useful in the field of psychiatry. Another example of a piperidine derivative is iloperidone (compounds 2, 7, and 8 in RF model run #2, Figure 4), which is an atypical antipsychotic for the treatment of schizophrenia. LIME highlighted the nitrogen atom of the piperidine ring. Amiperone (molecule 0 in ET model run #1, Figure 5) is a psychotropic and neuroleptic drug. LIME highlighted the piperidine ring in the amiperone molecule. Finally, the piperidine ring was also highlighted in spiroxatrine (molecule 6 in ET model run #2, Figure 5), which is used to treat psychotic disorders.

Piperazine derivatives were found twice by LIME: the anxiolytic drug enpiprazole (compound 1 in ET run #1, Figure 5) and the antihistamine drug chlorcyclizine (compound 3 in ET run #3, Figure 5). Finally, loxapine (compound 5 in RF model run #2, Figure 4) is used primarily in the treatment of schizophrenia. It is a member of the dibenzoxazepine class and structurally very similar to clozapine (compound 3 in ET model run #2, Figure 5). The piperazine substructure in both compounds is highlighted by LIME. On the other hand, LIME has highlighted a different substructure (the O=C–N–C=O ring substructure) than the piperazine ring in buspirone (molecule 6 in RF model run #3, Figure 4), which is a psychoactive drug used for the management of general anxiety disorders. The piperazine ring was also highlighted in several drugs to treat brain disorders: trazodone (compounds 1, 2, and 7 in ET model run #2, Figure 5), cloxypendyl (compound 1 in ET model run #3, Figure 5), and blonanserin (compound 2 in ET model run #3, Figure 5).

The aromatic nature of pyrazines and their potential interactions with the BBB components can also play a role in the permeability of compounds featuring these structures. Pyrimidines may also have implications for permeability, although the specific impact depends on the overall structure of the chemical compound. Phenothiazine derivatives are related to the thiazine-class of heterocyclic compounds that show antipsychotic activity for the treatment of anxiety disorders, depressive symptoms secondary to anxiety, and agitation. This class of compounds was found twice by LIME: trifluoperazine and imiclopazine were found in the output in the random tree models (triplicate in ET run #1, Figure 5, and triplicate in RF run #3, Figure 4) and the neural network model, respectively. Thioproperazine is another phenothiazine derivative used in the treatment of all types of acute and chronic schizophrenia. It was also found by LIME (compound 1 in DRN model run #2, Figure 3). Another good example is (E)-thiothixene (compounds 1, 2, 6, and 7 in RF model run #1, Figure 4), which is a typical antipsychotic of the thioxanthene class. It is also related to thioproperazine and pipotiazine, which are members of the phenothiazine class. LIME highlighted the same piperazine substructure in this class of molecules.

Another class of compounds is found in clothiapine. It is a typical antipsychotic of the dibenzothiazepine chemical class found by LIME (compound 2 in ET model run #1, Figure 5). Also, fasoracetam (molecule 2 in DRN model run #2, Figure 3) is a class of drugs that share a pyrrolidone nucleus that was not found by LIME. However, the other part of the molecule (piperidine ring) is highlighted by LIME. Another important example is menitrazepam (compound 7 in DRN model run #3, Figure 3), a hypnotic agent used to treat insomnia. It is a drug which is a benzodiazepine derivative. LIME highlighted the nitrogen-containing ring of this molecule. Understanding the diverse effects of nitrogen-containing heterocycles provides valuable insights for understanding the intricate landscape of BBB permeation.

Similarly, the hydroxyl group (−OH) is a crucial functional group because it can enhance permeability due to hydrogen-bonding interactions with the BBB components. Carbonyl groups (C=O) found in ketones and aldehydes can influence the permeability. The carbonyl oxygen can engage in interactions with the barrier components, affecting the overall transport process. For instance, pipamperone (duplicate compounds 0 and 2 in DRN model run #3, Figure 3) is a typical antipsychotic drug of the butyrophenone family used in the treatment of schizophrenia. LIME found that three substructures are important to its BBB penetration: the amide group, piperidine ring, and butyl ketone substructure highlights. The latter is part of the butyrophenone substructure, although the phenyl group was not highlighted. Esters (−COOR) and carboxylic acids (−COOH) can contribute to permeability, engaging in hydrogen bonding and other interactions and impacting the compound’s ability to transpose the barrier. Here, it is important to mention a specific, controversial example. Valproate pivoxil (compound 3 in DRN model run #2, Figure 3) is an anticonvulsant used in the treatment of epilepsy. It is likely a prodrug for valproic acid. LIME highlighted the −C(O)OCH_2_O(O)C– substructure as explanation for its BBB penetration. However, it is possible that the carboxylic group of valproic acid may be the reason for BBB penetration. Additionally, the size and charge of the functional groups play a pivotal role. Smaller and neutrally charged groups generally have a higher permeability. Other oxygen-containing functional groups also play a crucial role in influencing BBB permeability. Therefore, several key functional groups with oxygen have been identified for their potential to enhance permeability, as follows.

Compounds containing ether linkages (−O−) may exhibit favorable permeability characteristics. The oxygen in the ether group can participate in interactions that aid in the compound’s transposition across the barrier. For example, pinoxepin is an antipsychotic of the tricyclic group with a dibenzoxepin ring system that was found by LIME (compounds 3 and 4 in RF model run #2, Figure 4). Another example is the antiviral desciclovir (compound 6 in DRN model run #3 in Figure 3). It is a nucleoside analogue to purine and prodrug of the neurotoxic acyclovir with activity against viruses. LIME found that the purine side chain with oxygen-containing substructures (−O– and −OH) is highlighted, so the purine substructure may not be the explanation for BBB penetration. Understanding the nuances of these oxygen-containing functional groups is essential for designing compounds with optimized BBB permeability. As specific functional oxygen-containing groups can negatively impact the BBB permeation due to the reduction of the compound lipophilicity, it is essential to consider these intricate details in conjunction with the compound lipophilicity when evaluating its potential to permeate BBB.

With respect to the reduction of compound lipophilicity, it is important to mention that other functional groups also contribute to lipophilicity variations. For instance, halogen-containing compounds can enhance lipophilicity and, consequently, BBB penetration. Sulfur-containing groups such as thiol (−SH) can also influence lipophilicity, potentially affecting the compound’s ability to penetrate the BBB. The sulfur atom in thiol groups can form interactions with the BBB components, facilitating BBB penetration.

Compounds containing sulfoxide (−SO) and sulfone (−SO_2_) groups may exhibit enhanced permeability. Sulfonethylmethane (compound 9 in DRN model run #3, Figure 3) is a sedative-hypnotic and anesthetic drug with GABAergic actions. LIME highlighted the hydrocarbon substructure in the middle of the molecule instead of the sulfone group. Certain compounds with thiolsulfinate groups, characterized by a sulfur atom bonded to both oxygen and sulfur, can positively influence BBB permeability. By exploring the interactions of sulfur-containing functional groups, it is possible to gain valuable insights into designing compounds with improved capabilities for BBB penetration. Therefore, understanding the nuances of these groups is crucial to designing compounds with optimized characteristics for BBB penetration.

Halogen atoms (particularly bromine, chlorine, and fluorine atoms) can have a significant impact on BBB permeability. The presence of halogen atoms introduces unique characteristics that influence interactions with the barrier components. Fluorine atoms can enhance BBB permeability because fluorine is a small, electronegative atom that can influence the lipophilicity and electronic properties of the compound. Therefore, it potentially promotes favorable interactions for the BBB permeation. Similar to fluorine, the presence of chlorine atoms can alter the physicochemical properties of a compound, affecting its ability to penetrate the BBB. The larger size of chlorine compared to fluorine introduces specific steric and electronic effects that contribute to the overall permeation characteristics of the compound. Understanding the nuances of halogen-containing compounds is vital for medicinal chemists and researchers aiming to design drugs with improved blood–brain barrier penetration.

It is important to mention that corticosteroids penetrate the BBB, as presented in Figures 3–5. Our results found many corticosteroids such as diflorasone diacetate, triamcinolone benetonide, betamethasone dipropionate, fluocinolone acetonide, halcinonide, fluticasone propionate, paramethasone acetate, rofleponide, Mometasone, icometasone enbutate, and cortisuzol. Of all of these, cortisuzol is the only corticosteroid that does not have a halogen atom in its structure. LIME highlighted an oxygen-containing substructure to explain its BBB permeation for cortisuzol. The reader should note that, except for halcinonide, no corticosteroids had a halogen atom highlighted by LIME, indicating that they may only modulate the lipophilicity of the corticosteroids. Most corticosteroids had oxygen-containing substructures highlighted by LIME. In the pursuit of unraveling the mysteries surrounding corticosteroid penetration through the BBB due to its complex structure with halogen atoms and many cyclic oxygen-containing substructures, our latest research has illuminated crucial insights that beckon a re-evaluation of existing paradigms. Our study delves into the intricate relationship between corticosteroids and the BBB, shedding light on the pivotal role played by halogen atoms, specifically fluorine and chlorine atoms, as well as oxygen-containing groups. These molecular constituents emerge as potent modulators of corticosteroid lipophilicity, acting as gatekeepers that influence BBB permeability. Our findings underscore the significance of considering not only the structural composition and lipophilicity of corticosteroids but also the nuanced interplay of halogen atoms and oxygen-containing groups in dictating their ability to transpose the BBB. This revelation holds profound implications for drug design and development.

Also, some opioids were found in our study: levallorphan (compound 7 in ET model run #3, Figure 5), (−)-pentazocine (compounds 2 and 8 in RF model run #3, Figure 4), and cogazocine (compound 3 in RF model run #1, Figure 4). It is important to note that the nitrogen-containing substructure is explained by LIME as the cause for BBB penetration in two molecules, (−)-pentazocine and cogazocine. The two substructures highlighted in levallorphan are in the vicinity of the nitrogen-containing substructure that was explained as the cause of BBB penetration for the other two molecules.

Our results suggest that nitrogen atoms may have a special effect in facilitating the permeation of organic molecules, particularly when these nitrogen atoms might be protonated under physiological conditions. The studies conducted by Singh and colleagues^28^ and White et al.^30,31,41^ reveal certain substructures that are commonly found in compounds that permeate the BBB. These results support our study, as the presence of nitrogen-containing compounds and aromatic rings is found to be more prevalent in BBB-permeating compounds compared to nonpermeating compounds. Our study provides a comprehensive list of substructures that are crucial for BBB penetration, surpassing the existing knowledge obtained through explainable methods found in the literature to date.^28,30,31,41^ Although other functional groups such as oxygen-, sulfur-, and halogen-containing compounds play a role in regulating and balancing the lipophilicity of a compound, nitrogen-containing groups appear to be key in influencing compound permeability. Specifically, nitrogen-containing substructures such as carbamate, barbiturate, piperidine, piperazine, pyrazine, phenothiazine, dibenzothiazepine, benzodiazepine, butyrophenone, and dibenzoxepin are deemed more relevant to BBB permeation. However, both the lipophilicity and the charge of compounds need to be taken into account when determining the BBB permeation of a chemical compound. Corticosteroids serve as examples of the importance of lipophilicity. Additionally, opioid compounds appear to possess a nitrogen-containing substructure that is vital for BBB permeation.

### Re-Evaluation of the Methodology

K-fold cross-validation serves as a valuable technique for assessing the generalization performance of machine learning models on unseen data. It facilitates the optimization of model hyperparameters and aids in model selection for a given dataset. However, the use of the same cross-validation procedure and dataset for both tuning and model selection can lead to biased evaluations of model performance. Thus, the nested cross-validation approach is preferred to overcome this bias. This approach includes the hyperparameter optimization procedure within the model selection procedure, providing a less biased evaluation of the tuned models. The hyperparameters of most machine learning algorithms can be adjusted to fit a specific dataset. However, there are few guidelines for how to configure these hyperparameters. Instead, an optimization procedure is used to find the best hyperparameters for the dataset. This is where k-fold cross-validation comes in to select both the best hyperparameters for each model and the best model configuration.

K-fold cross-validation is effective for estimating model performance but has limitations. When used multiple times with the same algorithm, it can lead to overfitting. Each evaluation of a model with different hyperparameters provides information about the dataset. Noisy datasets tend to score worse, and this knowledge can be used to find the best configuration for the dataset. Although k-fold cross-validation attempts to reduce this effect, it cannot eliminate it completely. It is common for the hyperparameter optimization process to perform some form of hill-climbing or overfitting to the dataset. Nested cross-validation addresses the problem of overfitting the training dataset. Exposing the hyperparameter search to only a subset of the dataset provided by the outer cross-validation procedure reduces the risk of overfitting and provides a less biased estimate of model performance on the dataset. On the other hand, we chose to resample the imbalanced BBBP dataset using the standard Scikit-Learn library. The resampling method combined with 5-fold cross-validation was intended to combat overfitting instead of causing it. Overfitting occurs when a model learns the noise in the training data rather than captures the underlying pattern, resulting in poor performance on new data. The resampling technique aids in gauging the model performance on unseen data by iteratively training it on varied subsets of the dataset and assessing its performance on the remaining data. This iterative process offers a robust estimation of performance and guards against overfitting by promoting effective generalization to new data. Therefore, the resampling technique was instrumental in addressing potential overfitting issues.

The optimized parameters in the hyperparameterization process for the DRN, RF, and ET classifier models in three different runs for the blood–brain barrier permeation of compounds using the resampling method and 5-fold cross-validation are given in Tables S1 and S2. Table S3 shows the mean ROC-AUC of the training and validation data-sets for the BBB penetration model for three different runs using the DRN, RF, and ET classifier models for the blood–brain barrier permeation of compounds using the resampling method and 5-fold cross-validation. The precision, recall, F1, and accuracy scores and the Matthew correlation coefficient (MCC) for the DRN, RF, and ET classifier models are presented in Table S4 for three different runs for the validation dataset of blood–brain barrier permeation of compounds using the resampling method and 5-fold cross-validation. Figure S1 presents the confusion matrix for the DRN, RF, and ET classification models using the resampling method and 5-fold cross-validation of the BBB permeation. Figures S2–S4 show the most important substructures found by LIME for BBB penetration for the DRN, RF, and ET classification models using the resampling method and 5-fold cross-validation.

If the model performs significantly better on the training dataset compared to the validation dataset, this may indicate overfitting. Keeping this in mind, the results show that the ROC-AUC scores in the training dataset are not drastically greater than those for the validation dataset (see and compare Tables 3 and S3) before and after resampling. Therefore, that means the resampling method avoided overfitting. It was also found that the precision, recall, F1 and accuracy scores of the model on the training dataset are not significantly greater than those for the validation dataset (refer to Tables 4 and S4). Additionally, it is important to note that the accuracy ranged from 0.868 to 0.898. However, the Matthew correlation coefficient (MCC) on the validation dataset ranged from 0.645 to 0.699 due to the imbalanced nature of the BBBP dataset. MCC provides a measure of the quality of binary classifications based on the whole confusion matrix. It is considered a balanced measure and can be used even when the classes have substantial differences in sizes, as is the case here with the BBBP dataset. MCC ranges from −1 to +1, where +1 indicates a perfect prediction, 0 represents a random prediction, and −1 signifies total disagreement between the prediction and observation. While precision, recall, F1, and accuracy metrics offer valuable insights into various aspects of model performance, MCC is often preferred as it considers all elements of the confusion matrix and is especially useful with imbalanced datasets. It is worth mentioning that although the model was built using weights to address dataset imbalance, the MCC still only ranged from 0.645 to 0.699. Consequently, we opted to employ the resampling technique, resulting in an improved Matthew correlation coefficient on the validation dataset ranging from 0.850 to 0.947.

Another important point with respect to our methodology is that prior to parameter optimization, we divided our dataset into an 80:20 split. Subsequently, we utilized the training dataset, which consisted of 80% of the data, during the parameter optimization process. This approach ensured that the constructed model would not be overfitting. In addition to this, we performed 5-fold cross-validation and nested cross-validation for the purpose of comparison. Furthermore, we also resampled the imbalanced BBBP dataset, as previously explained. This led to even better results, particularly after resampling the BBBP dataset, but that does not mean the prior methodology produced overfitting.

The ROC-AUC scores in Tables 3 and S3 showed that the random forest and extra trees models do not overfit significantly, with scores around 0.99 and 0.91–0.94 for training and validation, respectively. The deep residual network (DRN) model had ROC-AUC scores ranging from 0.976 to 0.999 for training and 0.790–0.894 for validation, which is an indication of overfitting. However, our results on the imbalanced BBBP dataset were only reasonable for drug penetration (label 1) and poor for nondrug penetration (label 0), with an MCC score of only 0.65–0.70 (before balancing the dataset), as presented in Tables 4 and S4, although the precision, recall, F1, and accuracy for penetration were reasonable. After resampling the BBBP dataset, the model performed well for both labels, with MCC ranging from 0.90 to 0.95 using 5-fold cross-validation and 5 × 10-nested cross-validation (5-fold for inner and 10-fold for outer cross-validation) (see Tables 4 and S4). It is important to note that the ROC-AUC scores for training and validation of the model built with 5 × 10-nested cross-validation are around 0.999 and 0.988, respectively (similar results for 5-fold cross-validation) (see Tables 3 and S3). Once again, we have shown that both 5 × 10 nested cross-validation and 5-fold cross-validation with random forest and extra trees have similar results (see Tables S4 and S5) using the resampling method, do not overfit, and the MCC has considerably improved compared to the previous methodology. Therefore, we have decided to use the 5-fold cross-validation with the resampling method for both random forest and extra trees.

Figures S2–S4 present some of the known compounds with the most important substructures found by LIME for BBB penetration using the DRN, RF, and ET classification models, the resampling method, and 5-fold cross-validation: methylprednisolone succinate (a corticosteroid hormone (compound 0 in Figure S2 #1)); pirenzepine (a pyridobenzodiazepine (compound 3 in Figure S2 #1)); acetylmethadol (a diarylmethane and narcotic analgesic (compound 9 in Figure S2 #1)); fluorometholone acetate (a glucocorticoid (compound 0 in Figure S2 #2)); mazipredone and hydrocortisone aceponate (corticosteroids (compounds 3 and 4, respectively, in Figure S2 #2)); thiopropazate (a phenothiazine derivative (compound 6 in Figure S2 #2)); fluocinolone (a glucocorticoid (compound 0 in Figure S2 #3)); cortivazol (a steroid (compound 2 in Figure S2 #3)); chlorcyclizine (a phenylpiperazine (compound 4 in Figure S2 #3)); tybamate (a carbamate ester (compound 7 in Figure S2 #3)); mosapramine (an antipsychotic (compound 0 in Figure S3 #1)); sipatrigine (a member of pyrimidines and piperazines (compound 5 in Figure S3 #1)); triamcinolone diacetate (a corticosteroids (compounds 0, 3, and 7 in Figure S3 #2 and compound 2 in Figure S2 #1)); bromodiphenhydramine (an antihistamine (compound 4 in Figure S3 #2)); dixyrazine (a member of phenothiazines (compound 6 in Figure S3 #2)); fluocortolone and clobetasone butyrate (glucocorticoids (compounds 0, 1, and 2 in Figure S3 #3)); loprazolam (an imidazobenzodiazepine (compound 5 in Figure S3 #3)); oxymorphone (an opioid analgesic (compound 8 in Figure S3 #3)); flupentixol (an antipsychotic drug of the thioxanthene group (compounds 2, 4, and 6 in Figure S4 #1)); febarbamate (a member of barbiturates (compound 5 in Figure S4 #1)); amelometasone, Icometasone enbutate, and flumethasone (steroid hormones (compounds 0, 1, and 2, respectively, in Figure S4 #2)); nalmefene (a 6-methylene analogue of naltrexone, which is an opioid receptor antagonist (compound 3 in Figure S4 #2 and compound 3 in Figure S3 #3)); and clobetasol propionate and betamethasone benzoate (steroid compounds (compounds 0 and 1(8), respectively)).

### Limitations and Benefits

These structural explanations cannot be seen as rules, and the peculiar pharmacological properties of each molecule in the BBB permeability and the biology of the system must not be underestimated. Our focus is on creating structural interpretability from LIME and assessing whether the most important substructures to BBB permeability make sense with a human scientific understanding. To exemplify that these analyses are not well-defined rules but rather structural interpretations, some molecules such as steroid-like molecules in Figures 3–5 have polar groups, resulting in a higher polar surface area compared to the other molecules. Nevertheless, the steroid-like molecules are identified as permeable even though most of these molecules do not have nitrogen-containing groups. Indeed, it is important to admit that LIME may also make the wrong interpretations. For example, the maleate group highlighted in the teniloxazine maleate (compound 6 in run #2, Figure 3) is not the reason for BBB permeation. Another example of a limitation is related to the prodrugs. Triclofos (compound 3 in DRN model run #1, Figure 3) is a sedative drug used rarely for treating insomnia. It is a prodrug that is metabolized in the liver into the active drug trichloroethanol. Figure 3 shows the triclofos highlighted as the phosphate group, but it is not the active group. The same is true for petrichloral (compound 5 in DRN model run #1, Figure 3), which is a sedative and hypnotic chloral hydrate prodrug. However, we believe that interpretating substructures important to BBB permeability with a certain level of confidence can serve as a basis for analyzing counterfactuals and graphical neural networks, which are being increasingly explored for their ability to suggest structural changes in molecules to improve their permeability capacity.

Likewise, Lipinski’s rule of five^70,71^ states that a drug must have certain well-defined properties. Over the years, in recent science, drug discovery has been less and less adhering to these rules.^72^ A good drug for acting on the CNS must permeate the BBB. Studies show that it is easier to permeate with a smaller number of hydrogen bond donors and acceptors, for example. It is difficult to create a rule to predict whether a molecule could be a good drug. Consequently, the interpretation of substructures important to BBB permeability has been gaining prominence in the study of drugs such as toxicity and permeability in the BBB.

The skepticism around the effectiveness of LIME in helping researchers in the new synthesis of active molecules stems from several factors. First, designing new molecules with desired biological activity is a highly complex and multidimensional problem. While LIME can provide explanations for individual model predictions, it may not capture the full complexity of molecular interactions and structural requirements for activity. Second, the explanations of LIME are limited to local, instance-specific insights and may not provide a holistic view of the underlying structure–activity relationships crucial for molecule design. Third, interpreting results from LIME requires domain expertise in both machine learning and chemistry, making it challenging for researchers without a strong background in both fields to effectively utilize the method. Fourth, any insights provided by LIME would still need to be validated experimentally, which can be time-consuming and costly. While LIME can offer some insights into the predictions of machine learning models in drug discovery, its utility in guiding the synthesis of new active molecules may be limited due to the lack of experimental findings about structural alerts, and researchers should consider its potential drawbacks and limitations when incorporating it into their workflow.

Disregarding LIME and persisting in employing state-of-the-art techniques in drug research and discovery may inadvertently limit the understanding and interpretation of predictions generated by complex machine learning models. LIME presents several advantages over alternative, explainable artificial intelligence methods. These advantages include offering localized and instance-specific explanations, which are often more intuitive and easier to grasp compared to explanations at a global model level. Being model-agnostic, it allows its application to any machine learning model, regardless of complexity or underlying architecture. LIME generates explanations that are typically simpler, quicker, and more transparent than other methods, thus enhancing accessibility for nonexperts and fostering trust in model predictions. Identifying the significant features influencing individual predictions enables researchers to understand which features drive the model’s decisions. Additionally, it provides insights into complex models, such as deep neural networks, where traditional interpretability methods may face challenges. By integrating LIME, researchers can improve their ability to interpret and trust machine learning model predictions in drug research and discovery, leading to more informed decision-making and potentially accelerating the discovery of new drugs.

## Concluding Remarks

LIME was used to generate explanations regarding the permeability of organic compounds in the BBB. These explanations involve the substructures that contribute the most to the permeability of the molecule, or lack thereof, which serve as a useful guide for subsequent laboratory tests. LIME has a relatively fast execution time, with the large number of compounds in the dataset. It generates simple and intuitive explanations by highlighting molecules and indicating important substructures that facilitate BBB permeation. Understanding the important substructures that aid BBB permeation is vital for the pharmaceutical industry and for research groups involved in drug synthesis.

## Data Availability

Code, input data, and processing scripts for this paper are available at GitHub (https://github.com/andresilvapimentel/bbbp-explainer). The BBBP dataset cleaned by the authors is also provided on GitHub, together with the other data. The data analysis scripts of this paper are also available in the interactive notebook Google Colab.

## Supporting Information Available

Optimized parameters in the hyperparameterization process, the metrics (mean ROC-AUC) of the training and validation datasets for the BBB penetration model for the blood–brain barrier permeation of compounds using resampling method with 5-fold cross-validation; the precision, recall, F1, and accuracy scores, and the Matthew correlation coefficient (MCC) for the validation dataset of blood–brain barrier permeation of compounds using the resampling method with 5-fold cross-validation; and also with the nested cross-validation; the confusion matrix for the classification models using resampling method and 5-fold cross-validation; and the most important substructures found by LIME for BBB penetration using resampling method; 5-fold cross-validation; and the classification models (PDF)
