# Supplementary material for: Identifying Substructures That Facilitate Compounds to Penetrate the Blood–Brain Barrier via Passive Transport Using Machine Learning Explainer Models
Source: ACS Chem Neurosci. 2024 May 9;15(11):2144–59. doi: 10.1021/acschemneuro.3c00840 (PMC11157485; doi:10.1021/acschemneuro.3c00840)
Supplement: Supplementary file 1 — cn3c00840_si_001.pdf [file cn3c00840_si_001.pdf]

Identifying substructures that facilitate compounds to penetrate the blood-brain barrier via passive transport using machine learning explainer models

Lucca Caiaffa Santos Rosa, Caio Oliveira Argolo, Cayque Monteiro Castro Nascimento, and Andre Silva Pimentel*

Departamento de Química, Pontifícia Universidade Católica do Rio de Janeiro, Rio de Janeiro, RJ 22453-900, Brazil.

*Corresponding author: a_pimentel@puc-rio.br

Supplementary Material

Table S1. Optimized parameters in the hyperparameterization process for the Deep Residual Network (DRN) classifier model in three different runs (#1, #2, and #3) for the blood-brain barrier permeation of compounds using resampling method with 5-fold cross-validation.

Run	Layer size*	Drop out	Learning rate	Epochs	Momentum	Decay	Batches
#1	4	0.0	0.005	30	0.0	0.0	64
#2	4	0.0	0.005	20	0.9	0.0	32
#3	2	0.1	0.005	50	0.0	0.0	32

*Layer size: 2 for [256, 256], 3 for [256, 256, 256], and 4 for [256, 256, 256, 256]

Table S2. Optimized parameters in the hyperparameterization process for the Random Forest (RF) and Extra Trees (ET) classifier models in three different runs (#1, #2, and #3) for the blood-brain barrier permeation of compounds using resampling method with 5-fold cross-validation.

Model	Run	Depth	Estimators
RF	#1	19	323
	#2	19	300
	#3	19	305
ET	#1	19	112
	#2	18	359
	#3	19	490

Table S3. Metrics (mean ROC-AUC) of the training and validation datasets for the BBB penetration model for three different runs (#1, #2, and #3) using for the Deep Residual Network (DRN), Random Forest (RF), and Extra Trees (ET) classifier models for the blood-brain barrier permeation of compounds using resampling method with 5-fold cross-validation.

Model	Run	Training	Validation
DRN	#1	1.000	0.973
	#2	1.000	0.977
	#3	1.000	0.987
RF	#1	1.000	0.988
	#2	1.000	0.986
	#3	1.000	0.995
ET	#1	1.000	0.989
	#2	1.000	0.992
	#3	1.000	0.988

Table S4. The precision, recall, F1, and accuracy scores, and the Matthew correlation coefficient (MCC) for the Deep Residual Network (DRN), Random Forest (RF), and Extra Trees (ET) classifier models in three different runs (#1, #2, and #3) for the validation dataset of blood-brain barrier permeation of compounds using resampling method with 5-fold cross-validation.

Models	Run	Scores				
		Precision	Recall	F1	MCC	Accuracy
DRN	#1	0.952	0.952	0.952	0.906	0.952
	#2	0.924	0.924	0.924	0.850	0.924
	#3	0.935	0.935	0.935	0.873	0.935
RF	#1	0.947	0.960	0.954	0.908	0.954
	#2	0.934	0.968	0.950	0.902	0.950
	#3	0.966	0.983	0.973	0.947	0.973
ET	#1	0.948	0.972	0.959	0.919	0.959
	#2	0.966	0.972	0.968	0.936	0.968
	#3	0.950	0.957	0.954	0.908	0.954

Table S5. The precision, recall, F1, and accuracy scores, and the Matthew correlation coefficient (MCC) for the Deep Residual Network (DRN), Random Forest (RF), and Extra Trees (ET) classifier models in three different runs (#1, #2, and #3) for the validation dataset of blood-brain barrier permeation of compounds using resampling method with nested cross-validation.

Models	Run	Scores				
		Precision	Recall	F1	MCC	Accuracy
RF	#1	0.950	0.964	0.957	0.915	0.957
	#2	0.957	0.957	0.957	0.915	0.957
ET	#1	0.964	0.971	0.968	0.936	0.968
	#2	0.964	0.971	0.968	0.936	0.968

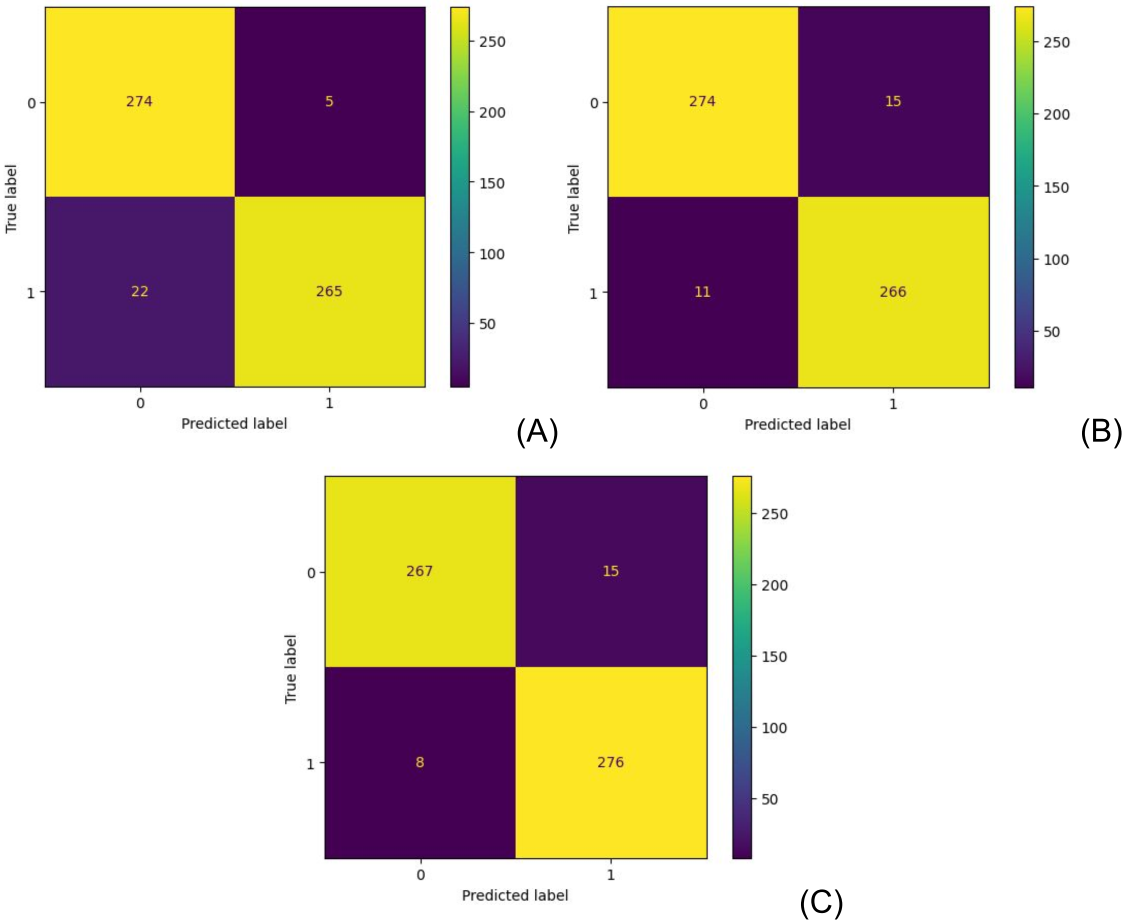

Figure S1. The confusion matrix for the DRN (A), RF (B), and ET (C) classification models using resampling method and 5-fold cross-validation of the BBB permeation for the runs #1.

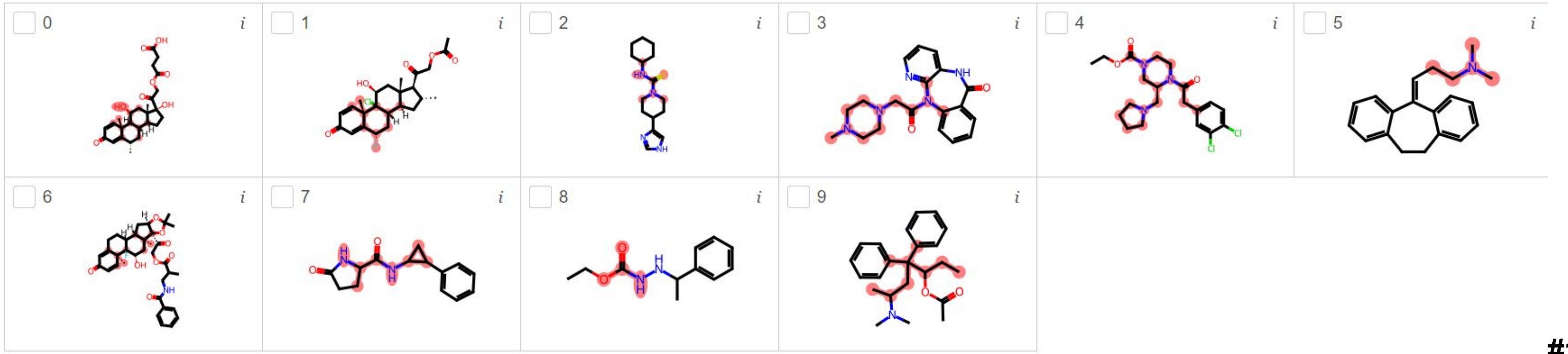

#1

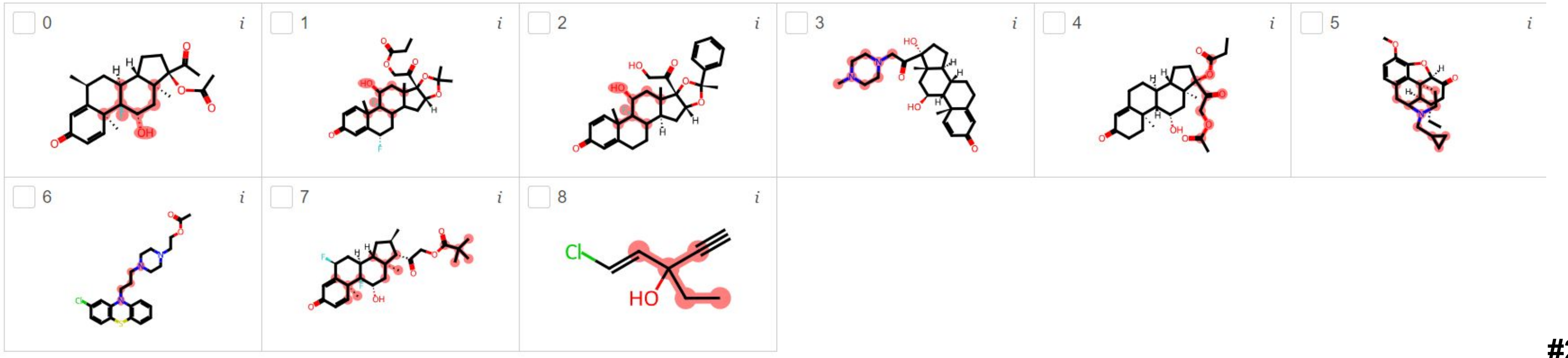

#2

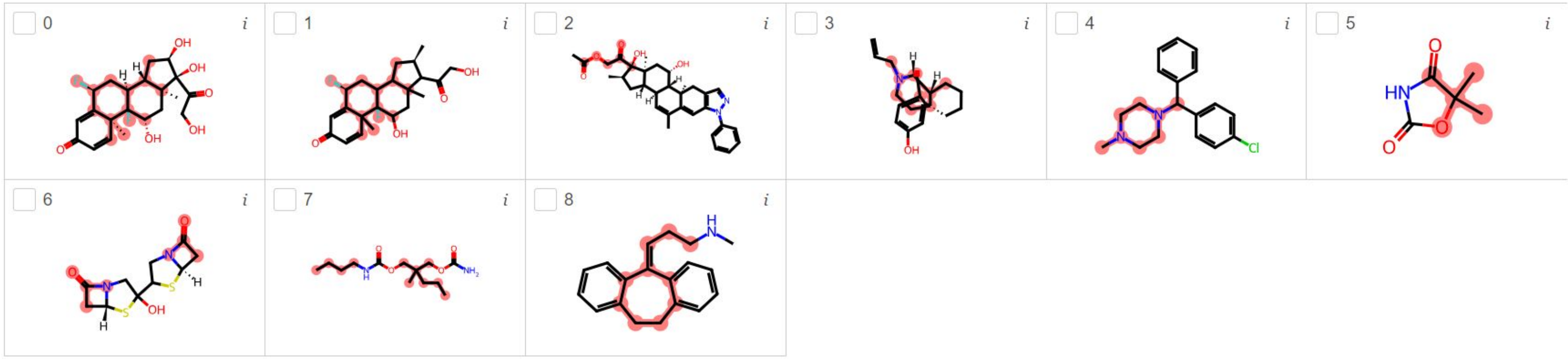

#3

Figure S2. The most important substructures found by LIME for BBB penetration using resampling method, 5-fold cross-validation, and the DRN classification model for runs #1, #2, and #3.

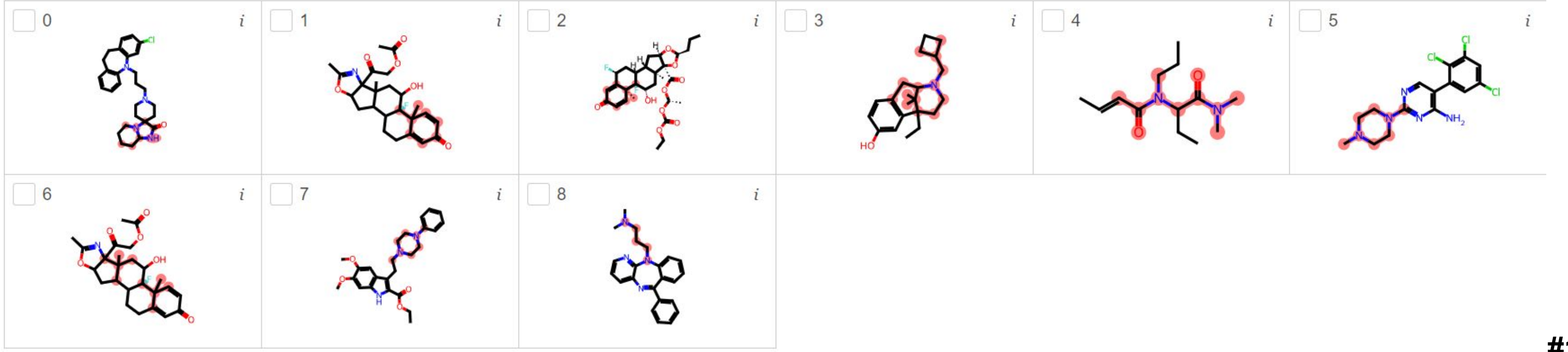

#1

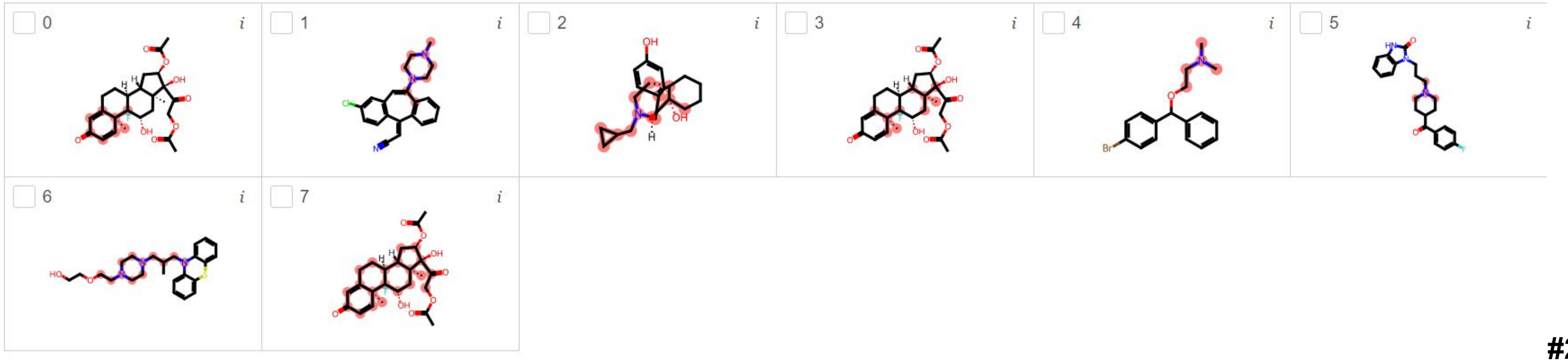

#2

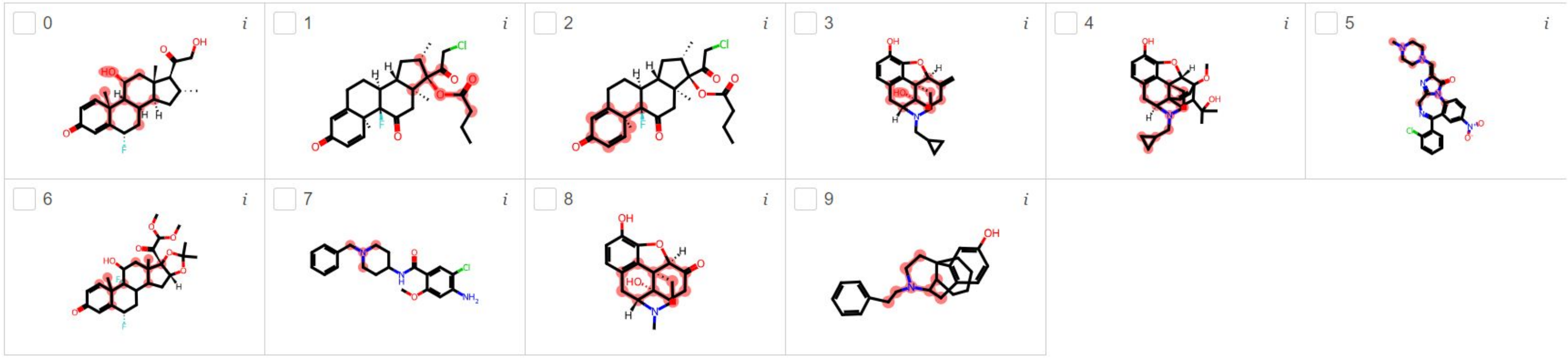

#3

Figure S3. The most important substructures found by LIME for BBB penetration using resampling method, 5-fold cross-validation, and the RF classification model for runs #1, #2, and #3.

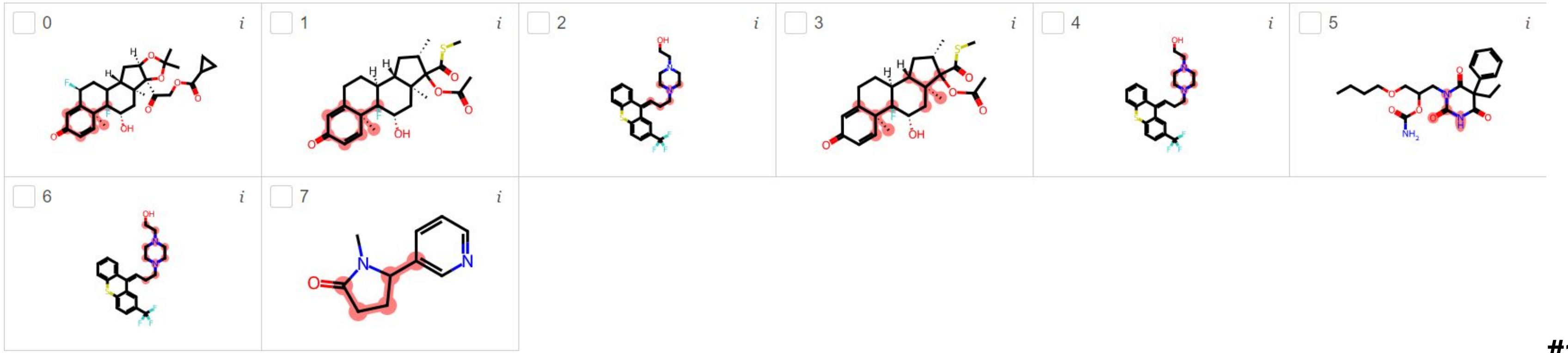

#1

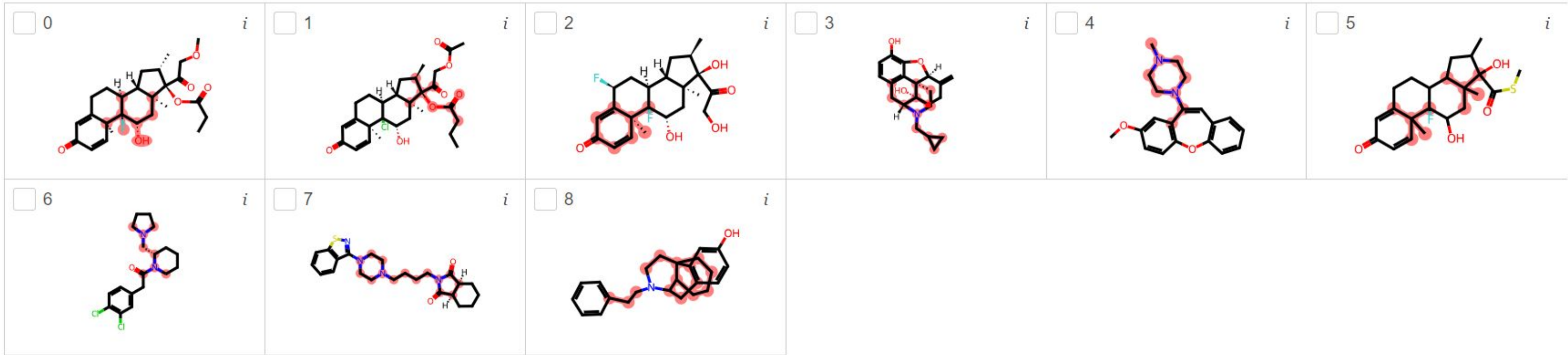

#2

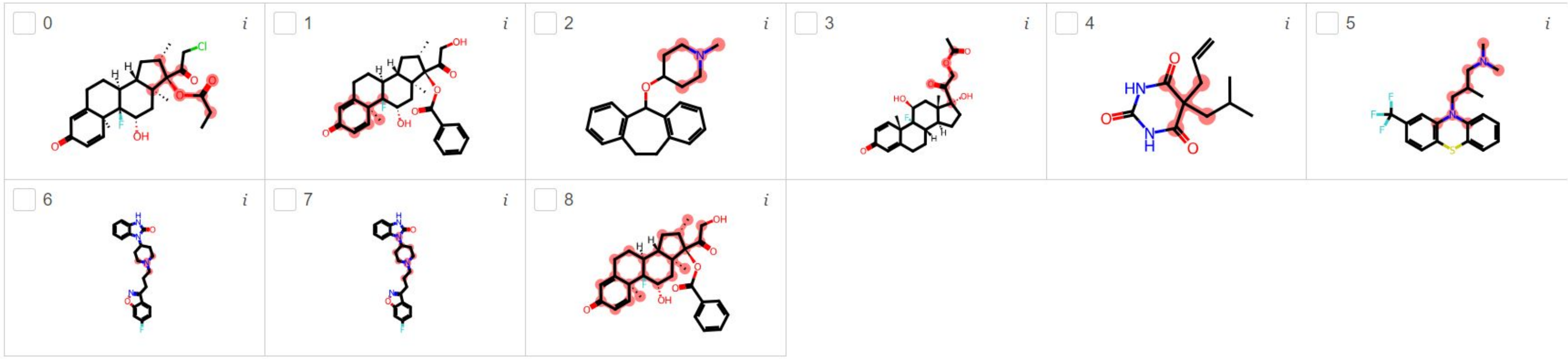

#3

Figure S4. The most important substructures found by LIME for BBB penetration using resampling method, 5-fold cross-validation, and the ET classification model for runs #1, #2, and #3.
